# Acute-Phase Inflammatory Reaction Predicts CMR Myocardial Scar Pattern and 2-Year Mortality in STEMI Patients Undergoing Primary PCI

**DOI:** 10.3390/jcm11051222

**Published:** 2022-02-24

**Authors:** Andras Mester, Nora Rat, Theodora Benedek, Diana Opincariu, Roxana Hodas, Monica Chitu, Imre Benedek

**Affiliations:** Department of Cardiology, George Emil Palade University of Medicine, Pharmacy, Science and Technology of Targu Mures, 540142 Târgu Mureș, Romania; andras.mester@yahoo.com (A.M.); theodora.benedek@umfst.ro (T.B.); diana.opincariu@umfst.ro (D.O.); roxana.hodas@umfst.ro (R.H.); iulia.chitu@umfst.ro (M.C.); imre.benedek@umfst.ro (I.B.)

**Keywords:** STEMI, primary PCI, inflammatory biomarkers, CMR, LGE, infarct size, transmurality, mortality

## Abstract

(1) Background: The inflammatory response following MI plays an important role in the healing, scar formation, and left ventricle (LV) remodeling. Cardiac magnetic resonance (CMR) imaging can accurately quantify the extent of myocardial scarring. The study aimed to investigate: (a) the relationship between acute inflammatory response and the CMR parameters of the scarring extent, and (b) the predictive power of inflammatory biomarkers and myocardial scarring for 2-year mortality. (2) Methods: The study included 202 STEMI patients, who underwent pPCI. Serum hs-CRP, IL-6, P-selectin, E-selectin, I-CAM, and V-CAM levels were determined at admission, and hs-CRP on the fifth day. Patients underwent LGE-CMR after 1 month, for LV volumes, ejection fraction (EF), infarct size (IS), and transmurality. Subjects were divided into tertiles according to the IS, and 2-year all-cause mortality was determined. (3) Results: IL-6 was associated with IS (r = 0.324, *p* = 0.01), increased transmurality index (r = 0.3, *p* = 0.01), and lower LVEF (r = −0.3, *p* = 0.02). Admission hs-CRP levels were not associated with IS, transmurality, or mortality, while hs-CRP at day 5 was a significant predictor for IS (AUC = 0.635, *p* = 0.05) as well as IL-6 levels (AUC = 0.685, *p* < 0.001). Mortality was significantly higher in the upper IS tertiles (6% vs. 8.7% vs. 24.52%, *p* = 0.005). IS was a significant predictor of 2-year mortality (AUC = 0.673, *p* = 0.002), with a cut-off value of 28.81 g, as well as high transmurality (AUC = 0.641, *p* = 0.013), with a cut off value of 18.38 g. (4) Conclusions: The serum levels of IL-6 and day-5 hs-CRP predict IS and transmurality, and day-5 hs-CRP levels are independent predictors of 2-year mortality in STEMI patients treated with pPCI. The CMR pattern of myocardial scarring after 1 month, as expressed by the magnitude of IS and transmurality, is a significant predictor for 2-year mortality after revascularized STEMI.

## 1. Introduction

Despite important advancements in interventional and pharmacological reperfusion treatment in recent decades, ST segment elevation myocardial infarction (STEMI) still remains one of the major mortality and morbidity causes in developed countries [1]. Long-term mortality rates range between 7% and 13% at 1 year and 8.5% and 23% at 2 years in patients who have undergone primary percutaneous coronary intervention (PCI) for STEMI [2,3,4]. Several biomarkers and scoring systems (e.g., TIMI, GRACE) have been developed for the prediction of short- and long-term adverse events and the mortality rates of these patients. However, none of these have demonstrated high accuracy in large clinical trials and meta-analyses [5,6]. Systemic inflammation has been validated as a major risk factor for atherosclerosis and an important promoter of plaque vulnerabilization, triggering acute coronary and cerebrovascular events [7]. Furthermore, inflammation plays an important role in the healing process following a myocardial injury. This process leads to myocardial scar formation with functional recovery or possible deleterious remodeling and subsequent dilation of the left ventricle (LV) [8]. Numerous biomarkers have been investigated in recent years for the evaluation of the inflammatory status in cardiovascular patients. High-sensitive C-reactive protein (hs-CRP) is currently considered a validated marker of systemic inflammation and is associated with atherosclerotic plaque progression, endothelial dysfunction, and acute coronary events. Additionally, it has also demonstrated a good predictive value for adverse events in patients with myocardial infarction (MI) [9,10,11]. Proinflammatory cytokine interleukin-6 (IL-6) has been associated with LV remodeling and adverse events in the post-MI period [12,13,14]. Local inflammation, mediated by cell adhesion molecules such as vascular cell adhesion molecule-1 (V-CAM-1), intercellular adhesion molecule-1 (I-CAM-1), and E- and P-selectin, also represents an important factor in the early myocardial healing process. In addition, adhesion molecules have shown elevated levels in patients following an acute coronary syndrome (ACS) compared to stable coronary artery disease patients [15,16]. Matrix metalloproteinases (MMP) are responsible for the extracellular matrix degradation of the myocardial cells following an injury and are associated with poorer outcomes in patients with ACS [17,18].

Cardiac magnetic resonance imaging (CMR) provides a uniquely accurate morphological and functional assessment by the precise determination of cardiac chamber volumes, masses, and wall motion abnormalities. Furthermore, tissue characterization is able to detect the extent of edema, inflammation, necrosis, and the viability of the myocardium following an ischemic injury [19,20,21]. Late gadolinium enhancement (LGE) sequences are the gold standard method for the quantification of myocardial scar tissue (infarct size), transmurality, and microvascular obstruction for each segment of the LV. Infarct size and the presence of microvascular obstruction determined at the acute phase is associated with poorer outcomes in patients with STEMI [22,23]. Recent clinical trials demonstrated that CMR findings can possess a better prognostic value compared to classic risk-stratification methods [24,25].

The aim of this study was to investigate: (a) the relationship between acute-phase inflammatory response and CMR parameters indicating the extension of the MI, and (b) the predictive power of inflammatory biomarkers and myocardial scarring for mortality at 2 years in patients who underwent primary PCI for STEMI.

## 2. Materials and Methods

### 2.1. Study Population

This prospective, observational study enrolled 202 patients admitted to the Cardiology Clinic of the Emergency Clinical County Hospital of Târgu Mures, Romania for ST-segment elevation acute MI, who were treated with primary PCI in the first 12 h after the self-reported onset of symptoms. The definition of the MI was in accordance with the fourth universal definition of myocardial infarction [26]. All patients received medical treatment according to the Guidelines of the European Society of Cardiology and hospital protocol. Patients with thrombolytic therapy, previously documented myocardial infarction, known active malignancy, life-expectancy under 2 years, autoimmune diseases, severe renal impairment (glomerular filtration rate < 30 mL/min/1.73 m^2^), acute or chronic infections at presentation, pericarditis, myocarditis, pregnancy, lactation, presence of devices/implants that were incompatible with magnetic resonance imaging, or known allergy to gadolinium were excluded from the study.

All patients signed the informed consent, and the study protocol was approved by the Ethics Committee of the Emergency Clinical County Hospital of Târgu Mureș and the George Emil Palade University of Medicine, Pharmacy, Science and Technology of Târgu Mureș. The study procedures were performed in accordance with the Declaration of Helsinki guidelines.

### 2.2. Study Procedures

#### 2.2.1. Clinical and Angiographic Characteristics

The medical history and demographic characteristics of all included patients were recorded. A 12-lead ECG and Killip class was recorded upon admission. The invasive coronary angiography data (infarct-related artery, pre- and postprocedural thrombolysis in myocardial infarction (TIMI) flow, multivessel PCI) were also collected.

#### 2.2.2. Serum Biomarkers

Venous blood samples were collected on the 1st and 5th day of admission and were stored at −80 degrees Celsius. The low-density lipoprotein (LDL), high-density lipoprotein (HDL) cholesterol, alkaline phosphatase, triglyceride, and N-terminal pro-B-type natriuretic peptide (NT-proBNP) levels were determined at the central laboratory of the Emergency Clinical County Hospital of Târgu Mureș. The inflammatory biomarkers were determined at the Center for Advanced Medical and Pharmaceutical Research of the George Emil Palade University of Medicine, Pharmacy, Science and Technology of Târgu Mureș: hs-CRP (COBAD Integra 400, Roche Diagnostics, Risch-Rotkreuz, Switzerland), determined on day 1 and 5; IL-6 (IMMULITE 2000 XPi, Siemens Healthcare GmbH, Erlangen, Germany); I-CAM and V-CAM (FLEXMAP-3D System, Luminex, Austin, TX, USA); MMP-9, E-selectin, and apolipoprotein b (Dynex Technologies, Chantilly, VA, USA).

#### 2.2.3. CMR Imaging

All patients underwent CMR examination at 1-month follow-up using a 1.5 T Siemens Magnetom Aera machine (Siemens, Erlangen, Germany). The following scanning protocol was used: left ventricle function and volumes were determined in cine mode in 10–12 short-axis steady-state free precession sequences, slice thickness 8 mm, field of view 400 × 400 mm, matrix 206 × 256, inversion time 17.5 ms, flip angle 59°. The acquisition of LGE sequences was performed at 10–15 min following intravenous administration of 0.1 mmol/kg gadopentetate dimeglumine (Magnevist, Bayer, Berlin, Germany). The postprocessing of the images was performed on QMass 8.1 (Medis, Leiden, the Netherlands) software with determination of LV ejection fraction (LVEF), indexed left ventricle end systolic and end diastolic volumes, myocardium mass and volume, stroke volume, and cardiac index. The infarct size was quantified in LGE sequences using delayed signal intensity analysis function with full-width half-maximum method in semiautomatic manner, with marking of the endocardial and epicardial borders. Transmurality was determined for every segment using the American Heart Association 17-segment model, and high transmurality was set at 50% for each segment.

**Figure 1:**
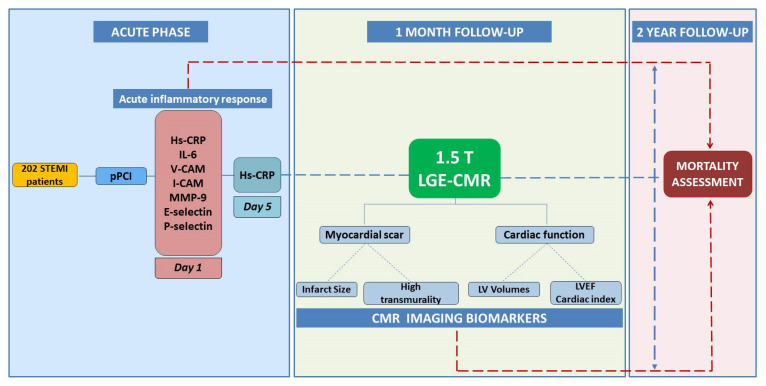
Flowchart of the study design and procedures.

The study subjects were classified into tertiles according to the infarct size (IS) mass, as follows: tertile I (IS < 14.79 g), tertile II (IS = 14.79–38.89 g), and tertile III (IS > 38.89 g). Patient follow-up was performed at 2 years from the acute event for determination of all-cause mortality rates. The flowchart of the study design is presented in Figure 1.

#### 2.2.4. Statistical Analysis

Statistical analysis was performed with GraphPad Prism 9.0.2 (GraphPad Software, San Diego, CA, USA) and ROC curve analysis was performed in MedCalc 20.013 (MedCalc Software Ltd., Ostend, Belgium). Continuous data variables are represented as mean ± standard deviation (SD) and qualitative categorical data are represented as numbers and percentages. Unpaired Student’s *t*-test was performed for continuous variables with Gaussian distribution and Mann–Whitney non-normal distribution. Correlations were computed using Spearman’s rank correlation test. One-way ANOVA test was performed for comparison of the three tertiles’ data. For calculation of adjusted odd ratios (OR) and 95% confidence interval (95% CI), univariable and multivariable logistic analysis was performed. The level of significance in this study was set at α = 0.05.

## 3. Results

### 3.1. Demographics and Clinical Characteristics

A total number of 202 STEMI patients treated with primary PCI in the first 12 h from symptom onset were included in the study. Based on the mean values of the IS mass determined at 1-month CMR follow-up, the patients were divided in three groups: tertile I with IS < 14.79 g (n = 50), tertile II with IS = 14.79–38.89 g (n = 102), and tertile III with IS > 38.89 g (n = 50). The mean age of the included patients was 62.99 ± 11.31 years, with no significant differences between the tertiles. The male gender was predominant in all tertiles, while hypertension and dyslipidaemia were the most frequently recorded risk factors, without statistically significant differences between the groups. The demographic data, pre-existing medical conditions, and risk factors are shown in Table 1.

### 3.2. Angiographic Findings

The left anterior descending artery (LAD) was most frequently identified as the infarct-related artery (IRA), with higher prevalence in tertile III. Patients in tertile III exhibited a significantly higher rate of Killip class ≥2 status (*p* = 0.048) at admission, suggesting more severe hemodynamic conditions in this category. Similar angiographic and procedural parameters were recorded in each tertile regarding stent implantation, multivessel PCI, and pre- and post-PCI TIMI flow. The angiographic and PCI procedure features of the patients are listed in Table 2.

### 3.3. Serum Biomarkers

The inflammatory response in the acute phase expressed by serum inflammatory biomarkers such as E-selectin, V-CAM, I-CAM, and MMP-9 determined on the 1st day of admission showed no significant differences between the three tertiles. In terms of the serum hs-CRP levels determined on the 1st and 5th day of admission, an increasing tendency was observed, without reaching statistical significance. However, the IL-6 serum levels determined on 1st day of admission were significantly higher in tertiles II and III (*p* = 0.002). NT-proBNP levels at admission were also significantly elevated in tertiles II and III (*p* = 0.033), as a surrogate parameter for heart failure. The lipid metabolic parameters were also similar in the three study groups. The serum biomarker parameters are summarized in Table 3.

### 3.4. CMR Parameters

The CMR imaging parameters determined at 1-month follow-up show a significantly increased LV myocardial volume and mass in tertiles II and III; however, the LV mass index (LVMI) to the body surface did not show significant differences between the groups. The extension of the myocardial scar tissue expressed by the infarct size determined in LGE sequences (mass and volume) was significantly higher in tertiles II and III (*p* < 0.0001). The percentage of the infarcted tissue of the LV was also significantly increased in the upper tertiles (8.01 ± 3.22 vs. 16.22 ± 4.98 vs. 26.35 ± 9.34, *p* < 0.0001). The transmurality of the myocardial scarring exhibited an increasing tendency from tertile I to III, with significant differences between the groups. On the other hand, the LV function quantified by LVEF was significantly lower in the tertiles with higher infarct size (*p* < 0.0001). The LV remodeling parameters—expressed by indexed ESV and EDV—were significantly elevated in the higher infarct size groups (*p* < 0.0001). The indexed LV stroke volume and cardiac index did not differ significantly between the three study groups. The CMR imaging parameters of the study groups are shown in Table 4.

No significant correlations were noted between the serum E-selectin and hs-CRP levels determined on the first day of admission and the CMR parameters determined at 1-month follow-up. On the other hand, day-5 hs-CRP levels significantly correlated with both the extent of the infarct size (r = 0.182, *p* = 0.047) and high transmurality zones (r = 0.225, *p* = 0.033). MMP-9 positively correlated with the LV infarct size (r = 0.319, *p* = 0.045). IL-6 also correlated with the infarct size (r = 0.324, *p* = 0.011), transmurality (r = 0.300, *p* = 0.019), and the percentage of the infarcted LV myocardium (r = 0.303, *p* = 0.018). A significant negative correlation was observed between the LVEF and day-5 hs-CRP (r = −0.234, *p* = 0.031), day 1 IL-6 (r = −0.305, *p* = 0.020), I-CAM (r = −0.116, *p* = 0.029), and V-CAM (r =−0.283, *p* = 0.029) serum levels. Adhesion molecules also negatively correlated with SVI (r = −0.021, *p* = 0.006 for I-CAM and r = −0.353, *p* = 0.006 for V-CAM) and cardiac index SVI (r = −0.003, *p* = 0.029 for I-CAM and r = −0.283, *p* = 0.029 for V-CAM). The correlations between the serum inflammatory biomarkers and CMR imaging parameters are summarized in Table 5.

### 3.5. Predictors of 2-Year Mortality

**Figure 2:**
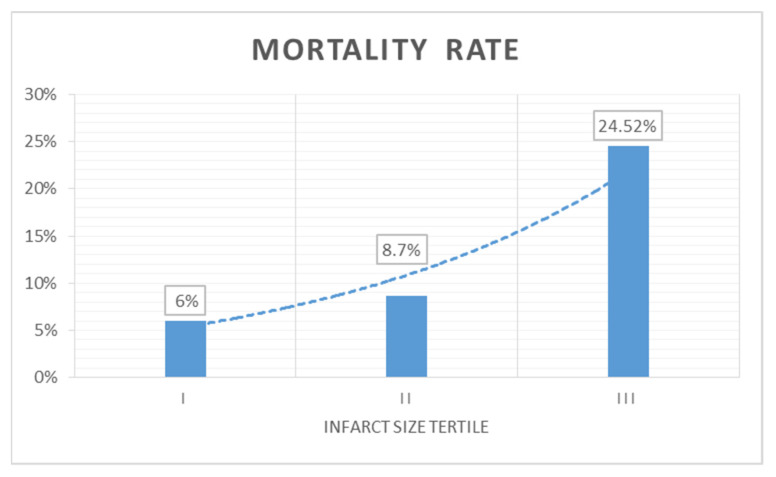
Two-year mortality rates and trend in the analyzed study group.

The overall mortality rate at 2-year follow-up in the study population was 12.8% (n = 26). Significantly increased mortality rates (*p* = 0.005) were recorded in tertile III (24.52%, n = 13), followed by tertile II (8.7%, n = 9), and with the lowest rates in tertile I (6%, n = 4). The mortality rates and trends between the tertiles are shown in Figure 2.

In addition, patients who passed away during the follow-up period were older (*p* = 0.03), with a significantly higher rate of diabetes (*p* = 0.008) and previous stroke (*p* = 0.005). Patients who passed away during follow-up also required more frequent multivessel PCI (*p* = 0.04), and a TIMI 3 flow was achieved at significantly lower rates (*p* = 0.008) compared to subjects who survived over the course of the two-year follow-up. The initial hs-CRP serum levels (determined on the first day of admission) were similar in the two groups (*p* = 0.057), and the hs-CRP levels determined during the 5th day after the acute event were significantly higher in the deceased group (*p* = 0.02). IL-6 levels were significantly higher in the deceased group (*p* = 0.02). Serum levels of E-selectin, P-selectin, V-CAM, I-CAM, and MMP-9 were not significantly different between patients who died and those who survived during follow-up. Both the LV infarct size (*p* = 0.006) and high transmural extent of the scar tissue (*p* = 0.006) were significantly higher in the deceased group, while the percentage of the infarcted LV myocardium was similar (*p* = 0.1). LV remodeling parameters including EDVI (*p* = 0.02) and ESVI (*p* = 0.01) showed significantly higher volumes, along with significantly reduced LVEF (*p* = 0.006) in the deceased group, while the global functional parameters, which included the SVI and cardiac index, were similar in patients who died and those who survived during follow-up. The serum inflammatory and CMR biomarkers of the two groups are summarized in Table 6.

**Figure 3:**
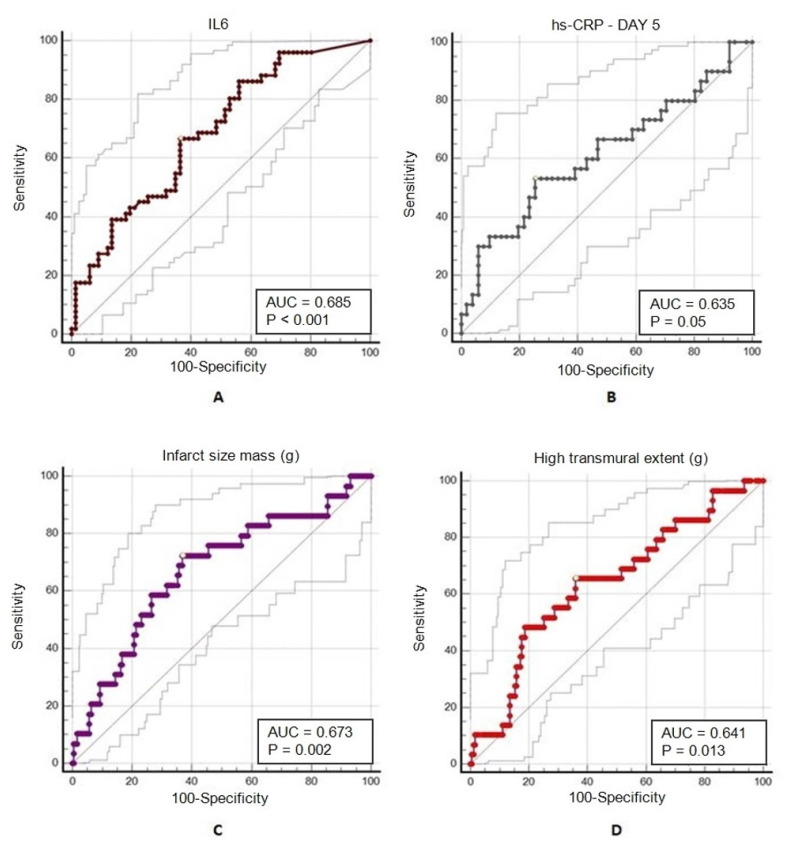
ROC curve analyses: prediction of infarct size based on (**A**) IL-6 and (**B**) day-5 hs-CRP; prediction of 2-year mortality based on CMR parameters (**C**) infarct size and (**D**) high transmural extent of the scar tissue.

A ROC curve analysis was performed for the evaluation of the predictive value of the serum inflammatory biomarkers and CMR parameters on the extension of infarct size and mortality. Serum IL-6 levels were identified as a strong predictor for the infarct size, with an area under the curve (AUC) of 0.685 (95% confidence interval of 0.593–0.768, *p* < 0.0002) and a cut-off value of >6.52 pg/mL, with 66.67% sensitivity and 19.7% specificity. Serum hs-CRP levels determined on the 5th day of admission were also established as predictors for LV infarct size, with an AUC of 0.635 (95% confidence interval of 0.504–0.724, *p* = 0.05) and a cut-off value of >5.7 mg/L, with 53.33% sensitivity and 1.96% specificity. The infarct size mass and high transmural extent determined at 1-month follow-up were predictors for 2-year mortality. An AUC of 0.673 (95% confidence interval of 0.60–0.737, *p* = 0.002) with a cut-off value of >28.81 g (72.41% sensitivity, 0.58% specificity) was identified for infarct size, and an AUC of 0.641 (95% confidence interval of 0.570–0.707, *p* = 0.012) with a cut-off value of >18.38 g (65.52% sensitivity, 1.44% specificity) for high transmural extent. Figure 3 presents the ROC curve analysis of serum inflammatory and CMR biomarkers’ potential as predictors of infarct size and 2-year mortality.

Multivariable analysis to predict infarct size detected that elevated serum levels of IL-6 (*p* = 0.04) are significant independent predictors for increased infarct size (of >38.89 g), after adjustments for day-5 hs-CRP, CMR-derived parameters, and clinical characteristics were performed (Table 7).

On the other hand, in multivariable analysis, the most powerful independent predictor for 2-year mortality was the serum level of hs-CRP (*p* = 0.004) determined on day 5, after adjustments for IL-6, CMR-derived parameters, and clinical characteristics were performed (Table 7).

## 4. Discussion

The current study aimed to investigate the relations between the acute inflammatory response and CMR parameters determined after 1 month, and their ability to predict mortality at a 2-year follow-up in patients undergoing primary PCI for STEMI.

There were no notable differences in terms of demographic characteristics or angiographic and PCI findings in the study population; however, a ≥2 Killip class at admission was more frequently present in patients with larger infarct size, as a consequence of larger myocardial damage, despite revascularization. Significantly increased NT-proBNP levels at presentation in the upper IS tertiles suggest the early presence of heart failure in patients with an extensive MI; however, its predictive power is limited in the first hours after ventricular dysfunction. Although the presence of diabetes, stroke, multivessel PCI, and a Killip class >2 was significantly higher in patients that did not survive the 2-year follow-up period, the multivariable analysis did not identify these as independent predictors for mortality. Similar observations were made regarding the postprocedural TIMI 3 flow, which was achieved at a significantly lower rate in the deceased group but was not identified as an independent predictor for mortality.

Our study evidenced that serum IL-6—as an important promoter of inflammation—levels determined on the day of admission showed an increasing trend through higher IS tertiles. Furthermore, significant correlations were identified with the extent of the myocardial scar tissue as expressed by both IS and high transmurality. In the SOLID-TIMI 52 trial, conducted by Fanola et al., which included 4939 ACS patients, the authors identified IL-6 as a predictor for adverse cardiac events at a median follow-up of 2.5 years [12]. Lino et al. evidenced an association between IL-6 and the development of heart failure following an AMI [27]. Although the current study did not identify IL-6 as a predictor for mortality at 2 years, it was found that patients who did not survive the 2-year follow-up period had significantly higher IL-6 levels determined at admission compared with the survivors. In addition, the multivariable analysis identified IL-6 as a significant independent predictor for LV IS. However, our study did not evaluate the presence of heart failure at 2-year follow-up, and IL-6 levels exhibited a significant negative correlation with the global LV function, expressed by EF, as early as one month following the MI.

Hs-CRP is a widely studied inflammatory biomarker, which has a proven predictive value in both chronic and acute cardiovascular settings. In a recently published study by Chen et al., which evaluated 3614 STEMI patients treated with PCI, the authors stated that postprocedural hs-CRP levels were associated with worse long-term outcomes. Patients with extremely low or high hs-CRP levels presented higher mortality rates at a median follow-up of two years [28]. Our results evidenced the fact that initial hs-CRP levels—determined on the day of admission, following the primary PCI procedure—were similar in each study group, regardless of the infarct size or transmurality, and were not associated with worse LVEF, remodeling parameters, or 2-year mortality rates. Nevertheless, the hs-CRP levels determined on the 5th day of admission positively correlated with the IS and transmurality of the myocardial scar tissue. The ROC curve analysis also identified day-5 hs-CRP levels as predictors for the IS and as being associated with reduced LVEF. These results confirm the observations by Al Aseri et al., who identified hs-CRP following a MI as a predictor for reduced LV function and heart failure [29]. Our previous results also evidenced the fact that patients with higher levels of serum hs-CRP in the acute phase of MI will exhibit larger IS and transmurality at 1-month follow-up [30]. However, though the initial hs-CRP levels of the 2-year survivors and deceased patients were similar, the 5^th^-day levels were three times higher in patients who were not alive at the 2-year follow-up. In addition, the multivariate analysis identified hs-CRP as an independent significant predictor for 2-year mortality. These data suggest that while the initial inflammatory response may not affect the long-term outcomes, the persistence of the inflammation may be responsible for worse outcomes. In a recently published meta-analysis, Kristono et al. suggest that a combination of biomarkers should be evaluated for better prognostic value and future inflammation mediatory therapies [31].

Other inflammatory biomarkers investigated in our study did not show predictive value for the extension of myocardial scarring or 2-year mortality. MMP-9 levels determined on the day of admission were correlated with the extension of the infarct size. In a recent study which investigated the role of inflammatory biomarkers in 1-year outcomes, Opincariu et al. identified MMP-9, I-CAM, and V-CAM as predictors for adverse events in STEMI patients [32]. In our study, I-CAM and V-CAM levels were negatively correlated with LV and global cardiac function expressed by LVEF, SVI, and cardiac index.

The meta-analysis of randomized trials postulated that infarct size determined with CMR is associated with mortality rates in patients undergoing primary PCI for STEMI. In addition, CMR-based scores have been developed for better risk stratification of STEMI patients [33,34]. Eitel et al. demonstrated that an IS determined with LGE that is greater than 19% of the LV is associated with adverse events at 1-year follow-up and possesses better prognostic value than clinical risk stratification or LVEF [35]. Our study results evidenced the fact that patients in the upper tertiles of IS had a significantly greater extent of high transmural scar tissue, as well as reduced LVEF and higher mortality rates. A significant increase in LV mass was observed in the upper tertiles, with greater IS and high transmural scarring. These findings were accompanied by a significantly increased IS expressed as the percentage of LV, suggesting that the increase in the IS mass and transmurality is not a result of the greater LV mass. Furthermore, the CMR parameters of LV remodeling, such as ESVI and EDVI, were markedly elevated, with an increasing trend in patients with greater IS, suggesting the presence of the onset of LV remodeling as early as 1 month following STEMI, while the global cardiac function expressed by SVI and cardiac index were still similar between the study groups. Additionally, IS and the extent of high transmurality scar tissue were identified as significant predictors for two-year mortality with cuff of values of >28.81 g for IS and >18.38 g for high transmurality. Patients who were deceased at 2-year follow-up had greater infarct size and high transmural extent, as well as greater indexed ESV and EDV, with reduced ejection fraction determined by CMR at 1-month follow-up. These data suggest that an early risk stratification of these patients can be performed based on the CMR imaging parameters.

The novelty of the current study derives from the multiomic approach that incorporates both inflammatory and imaging biomarkers for prediction of 2-year mortality in STEMI patients. The obtained results evidenced the impact of the acute inflammatory response on the extent of the myocardial scar tissue and LV remodeling parameters as well as on mortality.

### 4.1. Future Clinical Perspectives

The current body of evidence suggests that inflammation following an AMI plays an important role in the long-term outcomes of these patients, and several therapeutic modalities have been investigated in the last decade. Recent clinical trials assessed the efficiency and safety of systemic anti-inflammatory therapy in patients with acute myocardial infarction [36]. The investigators of the large, randomized, double-blind CANTOS trial, which included 10,061 patients with previous myocardial infarction, concluded that anti-inflammatory therapy with canakinumab was associated with significantly lower recurrent major cardiovascular events compared to placebo [37]. In another randomized, double-blind trial conducted by Tardif et al., 4745 patients were assessed within one month from a myocardial infarction and received anti-inflammatory therapy with colchicine versus placebo. At 2-year follow-up, the study concluded that low-dose (0.5 mg daily) colchicine was associated with a significant reduction in cardiovascular events compared to placebo [38]. Our study results also underline the importance of the inflammatory response on the long-term prognosis of STEMI patients, which can contribute to further research in this field.

### 4.2. Study Limitations

The current study excluded patients who did not survive until or did not present at the one-month CMR follow-up. The literature data show that early mortality in STEMI patients is mainly determined by complications that are linked to the magnitude of the MI. The aim of the study was to evaluate the long-term effects of inflammation and the extent of scar tissue to provide a better future prognostic tool for this patient category. Our study evaluated the all-cause mortality of the subjects and lacked data about cardiovascular death causes as well as the time of the occurrence of the death during the follow-up period. A further study will evaluate the incidence of major adverse events and additional CMR parameters with the development of an early risk-assessment system.

## 5. Conclusions

The inflammatory response in the acute phase of STEMI, expressed by serum levels of IL-6 and day-5 hs-CRP, is associated with the extent of the myocardial scar tissue and transmurality, as determined by CMR at 1-month follow-up. IL-6 levels are independent predictors for LV IS, while day-5 hs-CRP was identified as an independent predictor for 2-year mortality. Patients with larger infarct size have a greater extent of high transmurality scar tissue, larger LV volumes, and reduced LVEF. At the same time, CMR patterns of myocardial scarring at 1 month, as expressed by the magnitude of IS and transmurality, may predict 2-year mortality after revascularized STEMI. These results may provide insightful prognostic information for the risk stratification of STEMI patients.

## Figures and Tables

**Table 1:** Demographic information, medical history, and risk factors of the study population.

	Infarct Size I (<14.79 g) Tertile (n = 50)	Infarct Size II (14.79–38.89 g) Tertile (n = 102)	Infarct Size III (>38.89 g) Tertile (n = 50)	*p*-Value
Age (years ± SD)	64.31 ± 11.71	61.58 ± 11.29	63.08 ± 10.94	0.49
Height (cm)	168.2 ± 11.39	170.9 ± 10.2	167.58 ± 9.9	0.168
Weight (kg)	80.74 ± 13.21	85.30 ± 10.5	83.14 ± 11.75	0.378
BMI (kg/m^2^)	28.4 ± 4.28	28.7 ± 4.14	29.4 ± 3.45	0.124
BSA (m^2^)	1.88 ± 0.45	1.96 ± 0.81	1.92 ± 0.71	0.314
Male gender n (%)	32 (64%)	65 (65.7%)	40 (80%)	0.104
Hypertension n (%)	32 (64%)	60 (58.8%)	26 (52%)	0.39
Diabetes mellitus n (%)	6 (12%)	15 (14.7%)	5 (10%)	0.702
Documented CKD n (%)	3 (6%)	6 (5.8%)	3 (6%)	0.999
Current smoker n (%)	23 (46%)	39 (38.2%)	21 (42%)	0.651
Dyslipidaemia n (%)	17 (34%)	38 (37.2%)	25 (50%)	0.206
Stroke n (%)	3 (6%)	5 (4.9%)	7 (14%)	0.12
PAD n (%)	4 (8%)	9 (8.8%)	6 (12%)	0.759
Obesity * n (%)	20 (40%)	41 (40.1%)	18 (36%)	0.817
Prior PCI n (%)	5 (10%)	11 (10.7%)	4 (8%)	0.864

CKD—chronic kidney disease; PAD—peripheral artery disease, defined as ankle brachial index <0.9; BMI—body mass index; BSA—body surface area. * defined as BMI ≥ 30 kg/m^2^.

**Table 2:** Angiographic and PCI procedure characteristics of the study population.

	Infarct Size I (<14.79 g) Tertile (n = 50)	Infarct Size II (14.79–38.89 g) Tertile (n = 102)	Infarct Size III (>38.89 g) Tertile (n = 50)	*p*-Value
IRA				
LAD	21 (42%)	43 (42.1%)	27 (54%)	
CX	10 (20%)	16 (15.6%)	8 (16%)	0.528
RCA	19 (38%)	40 (39.2%)	13 (26%)	
LM	0 (0%)	3 (6%)	2 (4%)	
Killip class ≥2	10 (10%)	19 (18.6%)	18 (36%)	*0.048*
Stent implantation	48 (96%)	97 (95%)	48 (96%)	0.811
Multivessel PCI	17 (34%)	36 (35.2%)	18 (36%)	0.496
Pre-PCI TIMI flow 0–1	45 (90%)	89 (87.2%)	46 (92%)	0.117
Post-PCI TIMI flow 3	46 (92%)	95 (93.1%)	40 (80%)	0.120

IRA—infarct-related artery; LAD—left anterior descending artery; CX—circumflex artery; RCA—right coronary artery; LM—left main.

**Table 3:** Serum biomarker levels of the study groups.

	Infarct Size I (<14.79 g) Tertile (n = 50)	Infarct Size II (14.79–38.89 g) Tertile (n = 102)	Infarct Size III (>38.89 g) Tertile (n = 50)	*p*-Value
hs-CRP (mg/L)	6.60 ± 9.45	14.90 ± 33.87	14.67 ± 36.61	0.822
hs-CRP day 5 (mg/L)	17.01 ± 17.28	22.65 ± 16.73	27.31 ± 27.12	0.350
IL-6 (pg/mL)	9.17 ± 17.86	12.86 ± 18.61	13 ± 9.59	*0.002*
E-selectin (ng/mL)	64.31 ± 11.71	61.58 ± 11.29	63.08 ± 10.94	0.938
P-selectin	99.07 ± 96.78	96.44 ± 66.56	115.7 ± 101.5	0.518
V-CAM (ng/mL)	383.96 ± 504.5	447.67 ± 620.77	354.54 ± 579.12	0.792
I-CAM (ng/mL)	108.47 ± 217.09	175.50 ± 419.39	89.40 ± 154.75	0.648
MMP-9 (ng/mL)	451.6 ± 595.0	433.5 ± 566.3	324.8 ± 477.3	0.799
NT-proBNP (pg/mL)	270 ± 269.8	682 ± 624.1	1693 ± 2165	*0.033*
Apolipoprotein B (g/L)	0.99 ± 0.41	1.07 ± 0.37	3.59 ± 13.07	0.104
HDL chol (mg/dL)	57.38 ± 48.47	48.37 ± 39.38	55.48 ± 46.42	0.491
LDL chol (mg/dL)	103.3 ± 68.64	111.8 ± 68.49	129.6 ± 69.24	0.319
Triglycerides (mg/dL)	166.2 ± 111.5	169.7 ± 101.3	156.4 ± 89.4	0.117

**Table 4:** CMR imaging parameters of the study groups.

	Infarct Size I (<14.79 g) Tertile (n = 50)	Infarct Size II (14.79–38.89 g) Tertile (n = 102)	Infarct Size III (>38.89 g) Tertile (n = 50)	*p*-Value
LV myocardium volume (mL)	129.5 ± 32.89	159.2 ± 43.65	206.1 ± 47.85	*<0.0001*
LV myocardium mass (g)	127.1 ± 43.89	164.5 ± 49.58	216.4 ± 50.24	*<0.0001*
LV infarct size volume (mL) LV infarct size mass (g)	10.22 ± 2.90 10.37 ± 2.90	14.90 ± 33.87 25.50 ± 6.54	52.03 ± 17.16 54.65 ± 18.01	*<0.0001* *<0.0001*
LV infarct size percentage (%)	8.01 ± 3.22	16.22 ± 4.98	26.35 ± 9.34	*<0.0001*
High transmural extent (mL)	3.27 ± 3.39	14.65 ± 8.49	38.78 ± 24.86	*<0.0001*
High transmural extent (g)	7.07 ± 15.84	15.35 ± 8.89	39.83 ± 26.65	*<0.0001*
LVEF (%)	62.44 ± 9.59	53.17 ± 10.90	43.79 ± 10.22	*<0.0001*
EDVI (mL/m^2^) ESVI (mL/m^2^)	128.2 ± 46.28 55.11 ± 40.89	137.8 ± 36.05 71.37 ± 31.53	183.3 ± 47.46 103.5 ± 35.85	*<0.0001* *<0.0001*
SVI (mL/m^2^)	73.11 ± 13.52	70.98 ± 18.50	83.14 ± 24.10	0.069
Cardiac index (L/min/m^2^)	5.10 ± 1.64	6.0 ± 8.19	5.56 ± 1.83	0.287
LVMI (g/m^2^)	125 ± 35.98	123.3 ± 42.18	148.9 ± 40.05	0.019

LVEF—left ventricle ejection fraction; EDVI—indexed end diastolic volume; ESVI—indexed end systolic volume; SVI—indexed stroke volume; LVMI—indexed left ventricle myocardium mass.

**Table 5:** Correlations between the inflammatory serum biomarkers and CMR parameters.

	IL-6		hs-CRP Day 1		hs-CRP Day 5		MMP-9		I-CAM		V-CAM	
	r	*p*	r	*p*	r	*p*	r	*p*	r	*p*	r	*p*
LV myocardium volume (mL)	0.130	0.332	−0.042	0.692	0.161	0.130	0.130	0.291	0.118	0.457	−0.096	0.457
LV myocardium mass (g)	0.130	0.322	−0.042	0.692	0.161	0.130	0.130	0.291	0.118	0.457	−0.096	0.457
LV infarct size volume (mL) LV infarct size mass (g)	0.319 0.324	*0.013* *0.011*	−0.103 −0.103	0.333 0.334	0.182 0.182	0.047 0.046	0.319 0.324	*0.045* *0.044*	0.222 0.023	0.539 0.539	0.079 0.079	0.539 0.539
LV infarct size percentage (%)	0.303	*0.018*	−0.172	0.106	0.117	0.271	0.303	0.081	0.194	0.268	0.142	0.268
High transmural extent (mL)	0.300	*0.019*	−0.033	0.753	0.225	0.033	0.300	0.162	0.156	0.478	0.091	0.478
High transmural extent (g)	0.300	*0.019*	−0.035	0.744	0.223	0.035	0.300	0.154	0.159	0.481	0.091	0.481
LVEF (%)	−0.305	*0.020*	−0.152	0.166	−0.234	0.031	−0.305	0.312	−0.116	*0.029*	−0.283	*0.029*
EDVI (mL/m^2^) ESVI (mL/m^2^)	0.132 0.236	0.327 0.076	0.073 0.133	0.507 0.230	0.027 0.160	0.807 0.807	0.132 0.236	0.385 0.274	0.100 0.125	0.755 0.222	−0.041 0.161	0.481 0.755
SVI (mL/m^2^)	−0.186	0.166	−0.059	0.595	−0.112	0.310	−0.186	0.850	−0.021	*0.006*	−0.353	*0.006*
Cardiac index (L/min/m^2^)	−0.046	0.733	0.120	0.277	−0.010	0.924	−0.046	0.977	−0.003	*0.029*	−0.283	*0.029*
LVMI (g/m^2^)	0.023	0.863	0.151	0.172	−0.089	0.422	0.023	0.266	0.128	0.088	−0.223	0.088

r—Spearman correlation coefficient.

**Table 6:** Clinical information, PCI, inflammatory biomarkers, and CMR parameters in 2-year survivors and deceased patients.

	Alive n = 176	Deceased n = 26	*p*-Value
	**Demographics and Comorbidities**		
Age (years ± SD)	61.16 ± 11.36	67.73 ± 9.38	*0.033*
Male gender n (%)	101 (58.04%)	18 (69.23%)	0.832
Hypertension n (%)	101 (58.04%)	17 (65.38%)	0.677
Diabetes mellitus n (%)	18 (10.22%)	8 (30.76%)	*0.008*
Documented CKD n (%)	9 (5.11%)	3 (11.53%)	0.188
Current smoker n (%)	72 (40.9%)	11 (42.3%)	1.000
Dyslipidaemia n (%)	67 (38.08%)	13 (50%)	0.285
Stroke n (%)	9 (5.11%)	6 (23.07%)	*0.005*
PAD n (%)	14 (7.95%)	5 (19.23%)	0.077
Obesity n (%)	68 (38.63%)	11 (42.3%)	0.830
Prior PCI n (%)	17 (9.65%)	3 (11.53%)	0.727
	**PCI characteristics**		
Killip class ≥2	34 (19.31%)	13 (50%)	*0.0004*
Multivessel PCI	57 (32.38%)	14 (53.84%)	*0.046*
Pre-PCI TIMI flow 0–1	156 (88.63%)	24 (92.3%)	0.745
Post-PCI TIMI flow 3	162 (92.04%)	19 (73.07%)	*0.008*
	**Serum inflammatory biomarkers**		
hs-CRP day 1 (mg/L)	16.97 ± 48.31	20.69 ± 22.10	0.057
hs-CRP day 5 (mg/L)	14.74 ± 19.09	47.26 ± 48.55	*0.020*
IL-6 (pg/mL)	7.512 ± 4.67	17.55 ± 16.53	*0.029*
E-selectin (ng/mL)	71.25 ± 30.59	70.26 ± 20.44	0.806
P-selectin (ng/mL)	90.54 ± 70.82	96.54 ± 54.94	0.372
V-CAM (ng/mL)	444.51 ± 52.84	466.11 ± 71.74	0.928
I-CAM (ng/mL)	172.4 ± 85.97	133.7 ± 91.85	0.704
MMP-9 (ng/mL)	398.0 ± 41.54	274.0 ± 68.52	0.129
	**CMR parameters**		
LV infarct size mass (g)	28.17 ± 18.42	40.89 ± 21.31	*0.006*
LV infarct size percentage (%)	16.56 ± 8.70	20.07 ± 9.998	0.128
High transmural extent (g)	17.14 ± 18.60	27.98 ± 20.71	*0.006*
LVEF (%)	53.24 ± 11.92	43.62 ± 13.71	*0.006*
EDVI (mL/m^2^)	147.1 ± 46.35	196.5 ± 48.78	*0.020*
ESV I(mL/m^2^)	75.42 ± 37.28	122.0 ± 45.15	*0.015*
SV (mL/m^2^)	75.13 ± 20.70	74.67 ± 12.06	0.957
Cardiac index (L/min/m^2^)	5.03 ± 1.45	4.96 ± 1.08	0.984
LVMI (g/m^2^)	129.3 ± 40.12	166.7 ± 53.29	*0.033*

**Table 7:** Multivariable logistic analysis for prediction of IS and 2-year mortality.

**Multivariable Analysis for IS**			
**Variable**	**Adjusted OR**	**95% CI for ** **Adjusted OR**	***p*-Value**
hs-CRP day 5	0.5590	0.02916 to 3.370	0.5
IL-6	2.059	1.010 to 4.197	0.04
EDVI	1.02	1.00–1.05	0.1
ESVI	1.04	1.00–1.09	0.1
LVMI	0.98	0.95–1.00	0.2
LVEF (%)	1.00	0.90–1.11	0.3
Age	1.00	0.977–1.04	0.4
Diabetes	1.71	0.72–4.15	0.2
Killip class > 2	1.49	0.26–8.26	0.6
Multivessel PCI	2.50	0.93–7.14	0.07
Post-PCI TIMI flow 3	2.28	1.06–8.39	0.7
**Multivariable Analysis For 2-year mortality**			
**Variable**	**Adjusted OR**	**95% CI for adjusted OR**	***p*-Value**
hs-CRP day 5	13.75	1.8–23.7	0.004
IL-6	1.84	0.54–3.81	0.1
Infarct size mass (g)	0.97	0.80–1.17	0.7
High transmural extent (g)	1.04	0.93–1.18	0.4
EDVI	1.14	0.99–1.57	0.3
ESVI	0.84	0.50–1.06	0.4
LVMI	1.06	1.00–1.17	0.07
LVEF (%)	0.77	0.31–1.30	0.5
Age	1.05	1.00–1.12	0.05
Stroke	2.58	0.33–14.51	0.3
Diabetes	1.41	0.31–5.33	0.6
Killip Class > 2	1.61	0.07–13.39	0.6
Multivessel PCI	1.00	0.17–4.27	0.9
Post-PCI TIMI flow 3	1.58	0.49–9.36	0.4

## Data Availability

The data presented in this study are available on request from the corresponding authors. The data are not publicly available due to privacy and ethical restrictions (containing information that could compromise the privacy of the study subjects).
